# Effects of Timber Harvest on River Food Webs: Physical, Chemical and Biological Responses

**DOI:** 10.1371/journal.pone.0043561

**Published:** 2012-09-05

**Authors:** J. Timothy Wootton

**Affiliations:** Department of Ecology and Evolution, The University of Chicago, Chicago, Illinois, United States of America; Dalhousie University, Canada

## Abstract

I compared physical, chemical and biological characteristics of nine rivers running through three timber harvest regimes to investigate the effects of land use on river ecosystems, to determine whether these corresponded to changes linked with downstream location, and to compare the response of different types of indicator variables. Physical variables changed with downstream location, but varied little with timber harvest. Most chemical variables increased strongly with timber harvest, but not with downstream location. Most biological variables did not vary systematically with either timber harvst or downstream location. Dissolved organic carbon did not vary with timber harvest or downstream location, but correlated positively with salmonid abundance. Nutrient manipulations revealed no general pattern of nutrient limitation with timber harvest or downstream location. The results suggest that chemical variables most reliably indicate timber harvest impact in these systems. The biological variables most relevant to human stakeholders were surprisingly insensitive to timber harvest, however, apparently because of decoupling from nutrient responses and unexpectedly weak responses by physical variables.

## Introduction

Determining the effects of human activities such as land use on ecosystems is important for shaping management strategies and can also provide insight into the structure and function of ecosystems. The effects of human activity on river systems is often of particular interest because such activity can directly impact rivers through such mechanisms as water extraction, changes in flow regime and channel morphology, discharge of wastes, and changes in direct insolation. It can also indirectly impact rivers because of water flow through the surrounding watershed. The effects of logging on rivers, particularly coastal rivers of western North America, have recently attracted much interest because of their possible impacts on commercially important fish species such as salmonids. Much of the focus of logging impacts in these areas has centered on direct effects on fish via such mechanisms as temperature change [1], and changes in habitat structure relevant to spawning sites [2]–[8]. However, fish also depend on the food web in which they are imbedded, and logging may impact other components of rivers [9]–[12], altering sources and rates of energy input, primary and secondary production, predation risk, and prey taxonomic composition. Therefore, taking a more holistic view of river response to human activity may increase our understanding of the mechanisms of impact, which in turn may change our perspective on appropriate management strategies to take [e.g. 13], [14].

Beyond increasing our understanding of its effects, considering a range of potential ecological response variables may facilitate our ability to detect environmental change in the face of human activity. For example, several studies have suggested that variables such as changes in the abundance of different groups of species are more sensitive in detecting environmental impacts than are aggregate variables such as patterns of nutrient concentrations and physical conditions [15]–[17]. This result has not always been found [18], however, so further investigation of the issue is warranted to inform environmental monitoring strategies.

Systematic changes in river characteristics with downstream location [19] may provide some general preliminary predictions about the expected response of river food webs to logging. The source of energy input and consequent community structure from upstream to downstream reaches is expected to shift, driven by the expected increasing gradient of river width relative to the influence of the riparian zone vegetation. At upstream locations where the river channel is narrow, riparian vegetation can lean over the majority of the channel, shading out solar radiation and making leaf litter the dominant energy input. Therefore, upstream locations should be dominated by organisms that process large particles of leaf litter. At downstream locations, riparian vegetation is not capable of shading the entire channel and leaf litter introduced upstream has been broken down into small particles. Consequently, the ratio of new leaf litter input to algal-based production declines, and the food web is expected to be characterized by higher algal production and a shift in dominance toward animals which specialize as algal grazers or as collectors of small leaf particles. By removing riparian vegetation and leaf input from the surrounding forest, logging would be expected to disrupt this pattern, causing shifts in food web structure favoring organisms more typical of downstream reaches.

The role of nutrients in controlling river food webs may be important to consider, particularly in the context of differing land use regimes. For example, if production in rivers is nutrient-limited rather than light-limited, increases in solar radiation will not increase algal production. Nutrient loading may depend on logging and location within the river for several reasons [9]–[11], [20], [21]. For example, logging can have strong effects on nutrient flux into rivers because nutrient retention in watersheds and riparian zones by trees is lost [9], [22]. Logging can also have strong effects on the terrestrial species composition of the watershed, which in turn may have strong effects on nutrient cycling. In particular, forest succession along much of the west coast of North America proceeds from red alder (*Alnus rubra*), a deciduous species with N-fixing symbionts, to various conifer species. In logged areas, red alder becomes more dominant as the successional cycle is shortened, which may increase N-inputs into the watershed and shift the litter quality being added to the rivers [23]–[25]. Hence an increased understanding of nutrient concentration patterns and their impacts both with downstream location and in response to logging is desirable.

**Figure 1:**
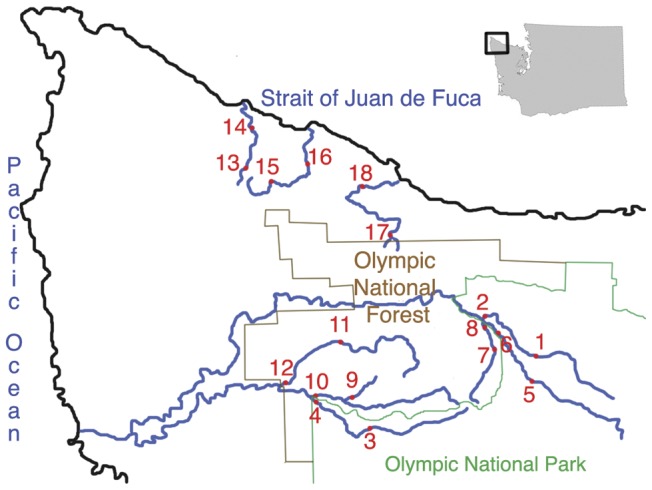
Location of study sites and land use boundaries on the Olympic Peninsula, Washington state, USA. Rivers: 1, 2-North Fork (N. F.) Sol Duc; 3, 4-South Fork (S. F.) Calawah; 5, 6-Main Stem (M. S.) Sol Duc; 7, 8-South Fork (S. F.) Sol Duc; 9, 10-Sitkum/Rainbow Creek; 11, 12-North Fork (N. F.) Calawah; 13, 14-Little Hoko; 15, 16-Clallam; 17, 18-South Fork/Main Stem Pysht.

Here I explore the response of river food webs to timber harvest on the Olympic Peninsula of Washington state, an area which has been a center of controversy over logging practices and river impacts, but for which there is surprisingly little published information on river structure and function. Because the Olympic Peninsula contains areas of substantially different timber harvest intensity in close proximity, ranging from national park to private timberlands ([26]; Table 1, Fig. 1), it provides a good situation to explore general patterns of change in river ecosystems with changes in land use. The goals of the study are 1) to document differences in river ecosystems in relation to timber harvest intensity, 2) to compare how different types (physical, chemical, biological) of variables respond to logging intensity, and 3) to explore how changes in river ecosystems correspond to differences in upstream/downstream location. I predicted that there would be strong responses of biological variables to timber harvest and downstream location, systematic shifts in nutrients levels with downstream location, increasing nutrient levels with increasing logging, shifts in nutrient limitation with downstream location, and a shift in the strength of nutrient limitation with increasing timber harvest, either because of higher light input (higher nutrient limitation) or higher nutrient input (lower nutrient limitation).

**Table 1:** Characteristics of sampling sites.

River (Land Holder*)	Stream Order	Elevation (m)	Source Distance (km)	Watershed Area (km^2^)	Time of Sampling
N. Fork Sol Duc, Up (NP)	2	487	14.16	61.5	1030
N. Fork Sol Duc, Down (NP)	3	346	22.37	80.7	1330
S. Fork Calawah, Up (NP)	2	223	14.81	40.1	1130
S. Fork Calawah, Down (NP)	4	127	24.62	59.9	1600
Main Sol Duc, Up (NP)	3	466	15.77	65.8	1130
Main Sol Duc, Down (NP)	3	347	25.11	106.1	1230
S. Fork Sol Duc, Up (NF)	2	420	9.17	16.5	1530
S. Fork Sol Duc, Down (NF)	3	352	11.75	31.1	1430
Rainbow Creek (Sitkum), Up (NF)	2	200	5.63	7.1	1430
Sitkum, Down (NF)	4	127	21.57	80.9	1530
N. Fork Calawah, Up (NF)	3	176	20.12	52.2	1700
N. Fork Calawah, Down (NF)	4	92	34.44	105.0	1700
Little Hoko, Up (NR)	2	179	2.25	3.5	1400
Little Hoko, Down (SP)	3	5	14.65	30.2	1000
Clallam, Up (NR)	3	122	6.12	9.4	1330
Clallam, Down (NR)	3	29	14.65	25.1	930
S. Fork Pysht, Up (P)	3	130	3.86	3.7	1600
Pysht, Down (P)	4	13	18.51	46.0	1600
P(Harvesting Association)	0.87	0.084	0.13	0.053	0.14
P(Downstream Location Association)	0.007	<0.001	0.001	0.002	0.85

* NP-Olympic National Park; NF-Olympic National Forest; NR-Washington Department of Natural Resources; SP-Washington State Parks, P-Private.

## Results

### Physical Variables

**Figure 2:**
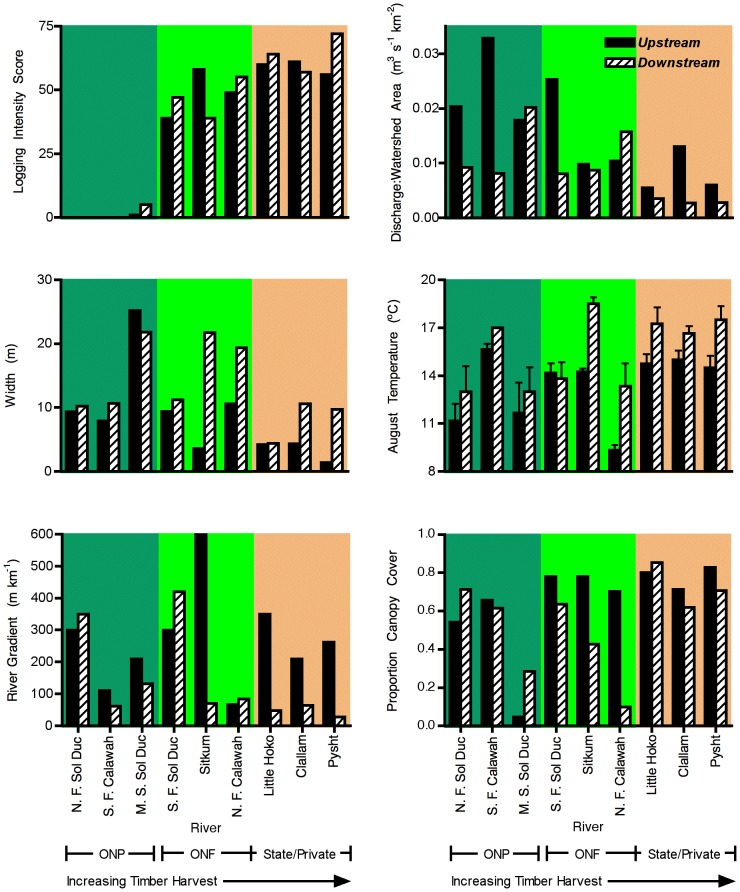
Variation in physical characteristics of study rivers with downstream location and harvest category. Variables included logging intensity, discharge per watershed area, width, temperature, river gradient, and canopy cover. Error bars ±1 s.e. for measures taken in multiple years. Physical characteristics tended to vary with downstream location but were inconsistently related to timber harvest.

Physical conditions generally tended to vary with downstream location, but were inconsistently related to timber harvest (Fig. 2, Table 1). As expected, timber harvest score increased when moving from national park to national forest to state and private lands. Stream order was higher with downstream location (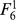 = 16.2, *p* = 0.007), but did not differ among timber harvest levels (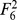 = 0.14, *p* = 0.87). River width, watershed area and discharge per unit watershed area jointly varied with timber harvest and downstream location (MANOVA, 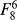 = 5.17, *p* = 0.019 and 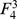 = 10.895, *p* = 0.021, respectively). In examining the individual responses with follow-up univariate tests, watershed area was necessarily higher with downstream location, and also showed a trend toward higher values for the unharvested rivers (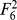 = 4.97, *p* = 0.053). River width increased downstream (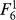 = 6.75, *p* = 0.04), but not with timber harvest (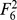 = 2.47, *p* = 0.17). Discharge per unit watershed area declined significantly both with harvest intensity (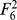 = 9.09, *p* = 0.015) and downstream location (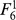 = 6.48, *p* = 0.04). River gradient was lower in downstream locations (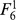 = 8.50, *p* = 0.03), but was not associated with harvest level (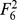 = 0.53, *p* = 0.61). Average temperature was higher in downstream than upstream sites (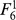 = 17.81, *p* = 0.006), but did not vary with timber harvest (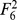 = 1.23, *p* = 0.36). This result did not arise from differences in sampling time among sites, as sampling time did not differ with either harvest level (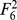 = 2.78, *p* = 0.14) or downstream location (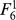 = 0.04, *p* = 0.85), and neither temperature, nor residuals of temperature from the model, were associated with time of sampling (*r* = −0.056 and *r* = 0.133, respectively; *p*>0.59). Canopy cover exhibited no main effects of downstream location (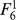 = 3.23, *p* = 0.12), or timber harvest (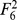 = 1.74, *p* = 0.25). There was a significant interaction in canopy cover and timber harvest, however (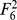 = 6.73, *p* = 0.029). Inspection of the data indicated that canopy cover tended to decline more strongly in downstream locations for rivers flowing through landscapes with intermediate harvest intensities (Fig. 2). No other land use by downstream location interactions were detected.

### Chemical Variables

**Figure 3:**
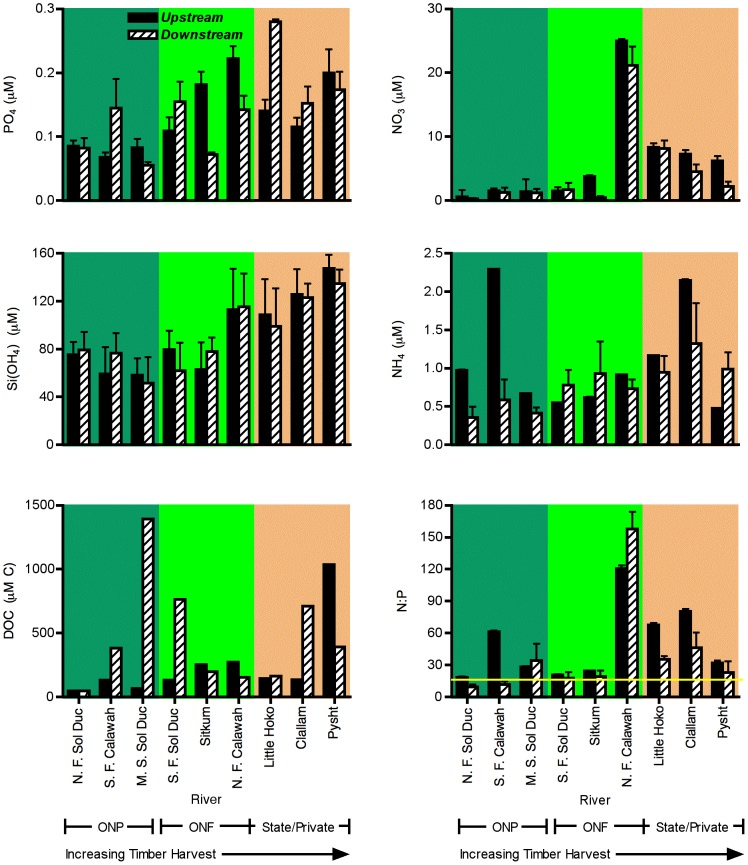
Variation in response of chemical characteristics of study rivers to downstream location and harvest category. Variables included phosphate, silicate, dissolved organic carbon, nitrate, ammonium, and N∶P ratio. Error bars ±1 s.e. for measures taken in multiple years. Chemistry tended to shift with harvest intensity but varied little with downstream location.

Chemical variables exhibited strong variation with harvest intensity, and little effect of downstream location (Fig. 3). Soluable reactive phosphate concentrations increased strongly with increasing timber harvest (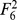 = 9.19, *p* = 0.023), but not downstream location (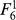 = 0.005, *p* = 0.95). Similarly, silicate concentrations increased markedly with timber harvest (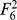 = 6.54, *p* = 0.03), but not with downstream location (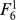 = 0.004, *p* = 0.95). Overall, nitrate and total nitrogen did not differ significantly with timber harvest (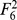 = 2.58 and 2.26, respectively, *p*>0.15). Nitrate also did not differ significantly with downstream location (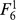 = 3.99, *p* = 0.09), but total nitrogen tended to decline with downstream location (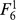 = 8.26, *p* = 0.03). Ammonium showed no significant variation either with harvest intensity (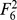 = 1.14, *p* = 0.38) or downstream location (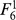 = 2.33, *p* = 0.18). Although nitrate and total nitrogen levels did not differ significantly with harvest intensity, visual inspection of the data suggested that there was a strong tendency to increase, and that one river, the North Fork Calawah, was a clear outlier (Fig. 3). When the North Fork Calawah was omitted from the analysis, both nitrate and total nitrogen showed significant increases with timber harvest (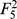 = 10.33, *p* = 0.02 and 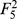 = 10.57, *p* = 0.02), and similar patterns with downstream location to the full dataset (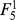 = 4.45, *p* = 0.09 and 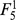 = 7.09, *p* = 0.05). Ratios of N∶P did not differ with either downstream location (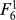 = 2.08, *p* = 0.2) or timber harvest (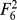 = 0.48, *p* = 0.64). In general, N∶P ratios suggested that P was most likely to be limiting, although values fell close to Redfield ratios in many cases. This observation also comes with the caveat that N and P co-limitation can occur over a range of N∶P ratios [27]. DOC did not vary significantly with either timber harvest (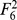 = 0.19, *p* = 0.83) or downstream location (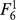 = 1.26, *p* = 0.31). There were no significant interactions between harvest intensity and downstream location detected for any chemical variables measured.

### Biological Variables

**Figure 4:**
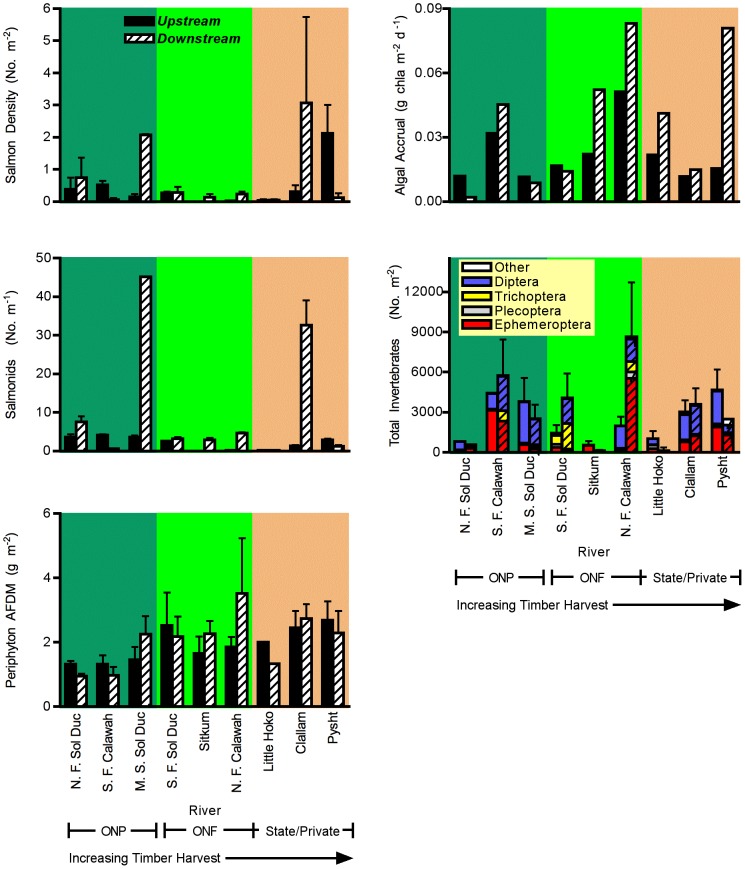
Variation in response of biological characteristics of study rivers to downstream location and harvest category. Variables included algal standing crop, insect abundance, salmonid density, salmonid abundance and algal productivity. Error bars ±1 s.e. for measures taken in multiple years. Biological variables showed inconsistent variation with land use and downstream position.

In contrast to physical and chemical variables, biological variables exhibited surprisingly little response to timber harvest or downstream location (Fig. 4). Algal ash-free dry mass (AFDM) did not vary significantly with harvest intensity (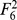 = 0.66, *p* = 0.20), or with downstream location (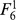 = 0.15, *p* = 0.71). Aquatic invertebrate abundance and composition did not vary significantly with either timber harvest (MANOVA, 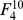 = 0.45, *p* = 0.86) or downstream location (MANOVA, 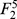 = 1.07, *p* = 0.55). Juvenile salmonid abundance, expressed either per unit area or per unit river length, also did not vary significantly with havest intensity (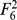 = 0.27 and 1.40, respectively, *p*>0.35) or with downstream location (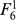 = 0.21 and 2.98, respectively, *p*>0.14). No significant interactions between timber harvest and downstream location were detected for these biological variables.

**Figure 5:**
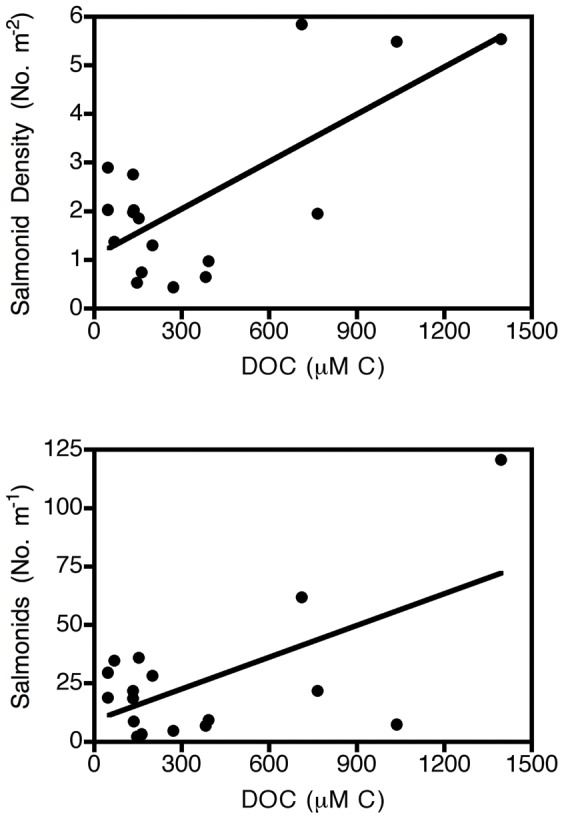
Relationship between dissolved organic carbon (DOC; µM C) and salmonid density (top) or numbers per meter of river (bottom). Lines of best fit from linear regression shown.

In the exploratory analysis of correlations between salmonids and other variables, few strong relationships were observed. One unexpected relationship that appeared was a strong positive association between juvenile salmonids and DOC (Fig. 5, density m^−2^: *r* = .717, *p* = 0.001, number per m river: *r* = 0.692, *p* = 0.007).

### Nutrient Limitation Experiments

**Figure 6:**
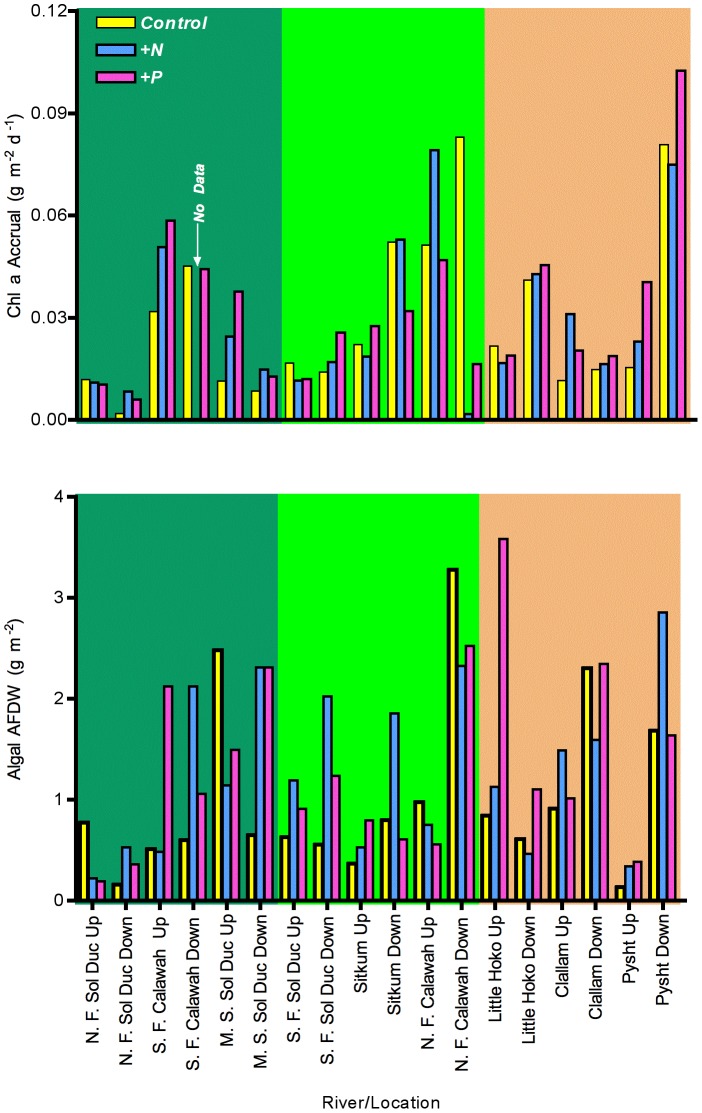
Periphyton accumulation in nutrient limitation experiments at each sampling site. Upper graph: nutrient diffusing glass discs. Lower graph: nutrient diffusing flowerpots. Experiments showed little evidence for consistent nutrient limitation with land use or downstream position.

Results from both types of nutrient limitation experiments were similar (Fig. 5). Algal production, estimated from algal accumulation for both flower pot (long-term) and porous disc (short-term) experiments (Fig. 6), did not vary with timbert harvest (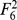 = 0.41 and 0.24, respectively, *p*>0.68) or downstream location (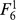 = 0.005 and 2.49, respectively, *p*>0.16). Additionally, nutrient additions caused no systematic change in algal production either in flowerpot (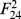 = 3.18, *p* = 0.06) or porous disc (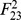 = 0.52, *p* = 0.6) experiments, nor were any statistically significant interactions found between nutrient treatments, timber harvest, and downstream location (all *p*>0.15), with the exception of one case: in the nutrient diffusing flowerpot experiment, there was a significant three-way interaction between nutrient treatment, timber harvest and downstream location (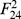 = 3.021, *p* = 0.04). Like most three-way interactions, interpreting this result is not straightforward: the effect appears to arise because both +N and +P treatments were higher than controls under no harvest, downstream conditions and under high harvest upstream conditions, but not in other situations (Fig. 6).

## Discussion

Physical variables measured in this study generally varied with downstream location as previously expected [19], with canopy cover generally declining, and water temperature,and river width increasing with downstream location in a river. Although physical changes in the river occurred with downstream location, the hypothesis that timber harvest would alter physical parameters in a similar manner was not generally supported. Temperature and river width did not vary with harvest intensity, nor was there an interaction between harvest intensity and downstream location [28]. Canopy cover also did not vary systematically with timber harvest, but an interaction between timber harvest and downstream location was detected. The pattern of this interaction did not provide strong support for the hypothesis, however, because it arose primarily from strong differences in canopy cover between upstream and downstream sites at intermediate, rather than high, land use intensities (Fig. 2), and because it was driven by substantially lower canopy cover at downstream harvested locations, rather than at upstream sites as was originally predicted. This pattern appears to be generated by unusually large variation between the sampling sites on the Sitkum and N. Fork Calawah in river width (Fig. 2), which is highly correlated with canopy cover (r = −0.91). Based on these results, it appears that current laws and management practices protecting riparian habitat from timber harvest have been largely successful, at least in the rivers selected for study in this relatively cool and overcast environment.

One physical factor that did change consistently was discharge per unit watershed area, which declined both with harvest intensity and downstream location. These patterns, which are opposite to those observed in other systems [29], [30], could arise for several reasons. First, higher accumulation of loose bed material under downstream or logged conditions might result in more subsurface flow, resulting in lower apparent discharge rates. Second, higher evaporation or transpiration in the watershed might occur, reducing discharge under both conditions. In the case of logged landscapes, such a circumstance could arise from increased irradiation and heating at the soil surface or shifts in vegetation from water-conserving conifers, which can effectively trap moisture in clouds and fog [31], to broadleaf trees, which have higher transpiration rates [32]. Such vegetation shifts were not observed in prior studies that found increased discharge in logged landscapes [29], [30], which might readily explain the difference in results. In the case of downstream position, more opportunity for direct evaporation from the stream with distance, perhaps in conjunction with lower gradient conditions (Fig. 2), might contribute to the pattern. Third, heterogeneity in precipitation input among and within watersheds might contribute to the pattern. Although the elevation of sampling sites did not differ significantly among logging intensities (Table 1), the upper reaches of watersheds, especially those with low logging intensity, tend to drain higher-elevation peaks in the Olympic Mountians. These peaks could induce additional precipitation when higher clouds are forced over them by prevailing onshore winds. Furthermore, these peaks are likely to receive a higher snowpack and maintian it into the summer, potentially resulting in additional input from snowmelt during the late summer sampling period.

In contrast to effects on physical variables, timber harvest had strong effects on water chemistry. Silicate levels, which likely correlated with sediment input rates [33], [34], increased significantly with land use as has been suggested in other studies of logging impact (1). Similarly, N and P levels were generally higher in areas of high harvest intensity, as has been documented in other watersheds [9], [10], [22], [35]. Higher P levels could have arisen from lower nutrient retention rates in harvested watersheds, or a higher interaction of water and P-containing soil and rock through higher sediment input. Higher N levels could have arisen from reduced nutrient retention in harvested watersheds or higher input in harvested watersheds dominated by N-fixing alder compared to intact watersheds dominated by conifers. The anomalously high N concentrations in the N. Fork Calawah also deserve some consideration. These concentrations were associated with substantial subterranean flow through a large section of the upstream riverbed. Studies in other rivers suggest that hyporheic microbial activity can have strong effects on nitrogen dynamics [36], [37]. Consistent with the hyporheic hypothesis, nitrogen concentrations in water sampled at a site just upstream of the area of complete subsurface flow were substantially lower (0.07 µM NO_3_, 0.03 µM NO_2_, 0.23 µM NH_4_) than at the primary sampling site downstream of the subsurface flow (26.7 µM NO_3_, 0.08 µM NO_2_, 0.92 µM NH_4_).

Water chemistry patterns did not support a hypothesis of systematic change along the river. In general, there was no effect of downstream location on any of the water chemistry variables examined. Given lack of experimental evidence for general nutrient limitation in these rivers, it seems unlikely that biological uptake could generate strong downstream patterns, but such patterns might appear in more nutrient-limited rivers [20]. Additionally, small tributary input into lower reaches, variation in local geology and strong effects of subsurface flow might have sufficiently important contributions to nutrient regimes to obscure any potential gradients.

In contrast to physical and chemical variables in the rivers, biotic parameters showed little consistent response to either timber harvest or downstream location. Salmonid density, insect density, algal standing crop and algal accrual did not vary with either harvest intensity or downstream location. The failure to find strong biotic responses to variation in timber harvest patterns is surprising given the strong responses of nutrients in the system. The failure of biotic variables to respond to harvesting might indicate high inherent variability on their part. These variables, however, readily responded to experimental perturbations of light [38, Wootton unpublished], flood disturbance [14], and riparian vegetation [Wootton unpublished] in other studies using similar methods and replication, suggesting that the observed lack of response may not be a problem of excessive variability, but of inconsistent response to harvesting in the study area. This result instead appears to arise because the biota apparently is not strongly limited by nutrients in these rivers. Using two different nutrient limitation experiments, there was little evidence that either N or P generally limited algal production in these systems. Because I did not include a +N+P treatment, it is possible that nutrient limitation was not detected because of co-limitation of algal production [27] coupled with a failure of algae to acclimate physiologically or to shift species composition in response to the altered N∶P input when single nutrients were added [39], [40]. Under the limited conditions where nutrient limitation seemed to occur (Fig. 6), both N and P addition treatments tended to be higher than controls, suggesting that nutrient co-limitation might be occurring, but that the algal community could shift its nutrient use to accomodate changing local N∶P ratios, so evidence of widespread nutrient limitation remains sparse.

Given recent concern about the effects of logging on salmonid populations, the failure to find strong negative responses of salmonids with increased land use is particularly surprising, and may suggest that other factors such as damming, water diversion, over-harvesting, changes in oceanic conditions and alterations in food web configuration have contributed more strongly to recent salmon declines. Several factors may act to minimize the generality of this result to other geographic areas, however. Specifically, the cool and relatively cloudy climate of the Olympic Peninsula may act to moderate the effects of logging activity on water temperature relative to other locations. Furthermore, riparian protection measures have been implemented in the study rivers that ran through higher land-use landscapes. These measures appear to be reasonably effective at reducing impacts from effects of logging activity, based on the lack of a relationship between several physical parameters that I measured and logging levels. Additionally, this study only examined juvenile salmonids rearing in rivers; salmonid species that spawn but do not rear in the rivers might be more adversely affected. Finally, this study provides a snapshot of river conditions at a particular point in time. It is possible that rivers running through high land use areas historically supported more salmon, but that relatively higher rates of salmonid decline are undetectable because of the absence of baseline data to compare pre-logging conditions to current conditions.

Although juvenile salmonid density did not vary consistently with harvest intensity and downstream location, it was associated strongly with one unexpected variable: DOC. Such an association could arise for at least three reasons. First, DOC is known to absorb UV radiation, and UV radiation can have negative effects on salmonids and other aquatic organisms under some conditions [41]–[43]. Hence DOC could promote higher salmonid populations. Second, DOC could be utilized by the stream biota as an energy source, leading to higher secondary production of salmonids. Third, migrating adult salmonids are thought to be important sources of carbon and nutrient import from ocean to river systems under some circumstances [44]–[47]. Consequently, river systems with high adult salmon migration might both contain more salmon offspring and higher carbon levels, leading to the association. This relationship requires further empirical exploration. For example, if the latter hypothesis were correct, then there should be a strong decline in DOC above versus below waterfalls that block fish movement.

In summary, patterns of change in rivers flowing through different timber harvest regimes did not follow predicted changes based on variation in downstream location. This discrepancy is perhaps in part explained by regulations designed to reduce logging impact in rivers, and also because some expected patterns with downstream location were not obtained. Alternative pathways of action could also have canceled out some of the expected responses [e.g. 12], [48], [49]. The response of physical and chemical variables in particular responded to timber harvest and downstream location in strikingly differences ways. It is also of interest to consider whether different types of ecological variables serve as reliable indicators of human impact. In their assessment of lake indicators, Cottingham and Carpenter [18] suggested that environmental indicators with high reliability, defined as low baseline variance and high responsiveness to environmental perturbations, were the most appropriate metrics to focus on. The results of this study might suggest that nutrient concentrations represent highly reliable indicators of logging impact, a feature observed in other areas too [9], [22]. Because different types of indicators respond differently to the same perturbation, however, consideration not only of the reliability of indicators, but also their relevance to the interests of stakeholders in these ecosystems may be important. In this case, although nutrient levels varied with harvest intensity, they did not exceed unsafe drinking water standards, and their use in detecting the existence of an environmental impact is limited because of the obvious visual nature of logging activities. In contrast, biotic variables relate directly to recreational and economic uses of the rivers, and therefore seem most important to incorporate into river monitoring schemes.

## Methods

### Ethics Statement

All necessary permits were obtained for the described field studies. With the exception of sites owned by Merrill and Ring (Pysht Tree Farm), all research was conducted on public lands. With the exception of salmonids, which were surveyed via visual observation, the research involved no endangered and protected species. The research plan was reviewed and approved by the Washington State Department of Fish and Wildlife, which determined that it did not require any permit. The study was reviewed and permits were provided by Olympic National Park, the Washington State Department of Natural Resources, and Merrill and Ring for research conducted on their lands.

### Study Sites

The study took place in rivers on the northwestern corner of the Olympic Peninsula (Fig. 1; Table 1). In this area, land use is primarily logging activity and occurs in three fairly distinct intensities [26]. No logging activity occurs within Olympic National Park, moderate logging activity occurs within Olympic National Forest, and fairly intense logging activity occurs on both private and state forestlands.

Study rivers and sites were selected using topographic maps and maps of land ownership, based on the general land use categories previously documented (national park, national forest, state/private land), the existence of access points from roads or trails, and the likelihood that sampling equipment would not be disturbed by curious visitors. Three rivers were selected in each timber harvest category (Fig. 1), and two reaches within each river were chosen as sampling sites to produce a split-plot design (rivers nested within harvesting categories, upstream-downstream location nested within rivers). The study rivers within Olympic National Park were the North Fork Sol Duc, the main stem Sol Duc, and the South Fork Calawah. Rivers within Olympic National Forest were the South Fork Sol Duc, North Fork Calawah, and Sitkum. Rivers on state and private land were the Clallam, Little Hoko, and Pysht. Distances between sites within a river averaged 9.0±3.9 km, ranged between 2.6 (South Fork Sol Duc) and 14.6 (Pysht) km (Fig. 1), and did not differ among timber harvest levels (ANOVA, P = 0.61). To increase comparability among rivers, sampling sites were chosen in run habitats flowing out of pools, characterized by approximately laminar flow and cobble substrates ranging from 4–15 cm diameter. These habitats are also heavily used by juvenile salmonids in these study rivers. I did not include a larger number of rivers both because of logistical limitations, and because I wanted to minimize the introduction of spatial variability in environmental conditions (climate, glacial runoff, etc.), thereby maximizing the chances of detecting a harvesting effect.

Variation in timber harvesting intensity across the land ownership categories, which was previously documented [26], was confirmed for the study watersheds by analyzing satellite photographs of the watersheds taken circa 2002, which were downloaded from Google Maps® in August 2005. Watersheds were delineated with the aid of topographic maps, and a grid of points was placed over each watershed image using Image J software. At each point (minimum 25 per watershed), the land was scored as either alpine (*Alp*), mature conifer (*MC*), alder/young conifer (*AYC*), or recently harvested (*RH*; no tree canopy but below treeline). I then derived a logging intensity (*LI*) score for each watershed using the following formula which is scaled by typical successional ages in years of each habitat type:
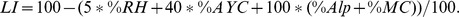
This index ranges from 0 (minimal logging for the past 70 or more years) to 95 (completely logged in the past 10 years). An potential alternative index, the primary axis in a principle component analysis of non-alpine habitat variables (accounting for 73.1% of variation in habitat), correlated highly with the logging index (*r* = −0.998).

### Sampling of Variables

At each site, the rivers were sampled for a variety of physical, chemical and biological variables. Sampling took generally took place between May and early September between 1997 and 2002, and samples from all sites were collected within a five day period. Temperature was sampled during August with a thermometer placed on the river bottom for 5 minutes. Because time of day when sampling occurred necessarily varied, I checked for a relationship between average temperature and time of sampling and found none (*r* = −0.056). Width was measured with tape measures and depth and current velocity were measured at several points across the site with a flow probe (Global Water) located at 60% of the maximum depth below the surface. Discharge was calculated as width×average depth×average velocity during summer base flow. River gradient (m drop per km length) and stream order were estimated at each site from United States Geological Survey topographic maps (1∶24000 scale). Canopy cover, used as an index of leaf and light input, was measured with a spherical densiometer by two observers in each of four directions (directly upstream, directly downstream, left bank and right bank). With the exception of temperature, which was taken at sampling visits in 1997, 1998 and 1999, other physical measurements were taken in 1997, although additional measures were retaken on occasion in subsequent years to verify results which appeared anomalous (e.g., lower downstream discharge, higher downstream canopy).

Nutrients were sampled by collecting water with a 50 cc syringe and filtering the water into acid-washed, numbered Nalgene bottles through a GF/F syringe filter to remove micro-organisms. Samples were kept cool and frozen upon return to the laboratory. We collected nutrient samples in early August 1998 and 1999, in late July 2001, and in late May 2002. Frozen samples were express-shipped to the University of Washington Marine Chemistry Laboratory, where they were analyzed for PO_4_ (SRP), SiOH_4_, NO_3_, NO_2_, and NH_4_ following the procedures outlined in [50]. In 2001, additional water samples were collected for analysis of DOC following similar procedures.

Biological variables sampled included periphyton accumulation and biomass, invertebrate populations and fish densities. Periphyton standing crop was sampled in September 1997, August 1998, and August 1999 by placing 7.5×7.5 cm ceramic floor tiles on the cobble bottom and allowing them to incubate for 2 months [10], [42]–[44]. Periphyton material was scraped from the tiles, homogenized with a blender, filtered through glass-fiber filter paper (GF/C), dried at 70°C for 24 hours, weighed, combusted at 500°C for 4 hours, and reweighed to calculate algal ash-free dry masses. Invertebrates were sampled in September 1997 and August 1999 by placing two 15×15 cm ceramic floor tiles on the cobble bottom to serve as standardized sampling substrates similar to cobbles [14], [51]–[53]. After a 2 month incubation, tiles were collected in a fine-mesh dip net, and invertebrates were dislodged and filtered into a “Parker filter”, a 0.05 mm^2^ mesh filter made from no-seeum netting sandwiched between the lid and rim of a 1 liter plastic storage jar with the jar bottom and the lid center removed. Samples were stored in 70% Ethanol and enumerated under a dissecting microscope in the laboratory, where individuals were identified to family.

To sample fish, four 60×60 cm quadrats were placed on the river bottom, and after a 5 minute waiting period to allow fish to return to their normal behavior, the number of fish either present in the quadrat at the start of the census, or which entered the quadrat over the subsequent 5 minute period were counted by a motionless observer standing nearby. If no fish was counted in the first four quadrats, additional quadrats were censused using the same procedures until a fish was recorded. Fish were sampled in September 1997 and August 2001. This relative measure of fish activity was calibrated to estimate instantaneous fish densities by conducting censuses in areas of varying fish density along the S. Fork Pysht River and simultaneously recording fish densities nearby with time-lapse underwater video equipment. Videotaped densities of fish in a 0.2 m^2^ area were counted at 1 minute intervals and the average instantaneous densities compared to quadrat censuses taken simultaneously from other locations in the study reach using linear regression. From this analysis, the equation which best fit the data (*r* ^2^ = 0.942, *n* = 10, *p*<0.0001) was: *Fish Density (m^−2^) = 0.228·Quadrat Count*. Fish were analyzed both on a density per unit area basis, and on a number per unit river length (density×river width) basis. This quadrat method was chosen over others because it is logiscally easy to implement in remote sampling sites, has a lower likelihood of altering fish behavior than other passive methods (e.g. snorkeling), and was more acceptible to several landholders who were reluctant to permit methods that could potentially harm or kill fish (e.g., electroshocking or rotenone addition).

### Nutrient Limitation Experiments

Because I hypothesized that nutrient concentrations would vary with downstream location and land use, I conducted experiments testing for nutrient limitation at each site. Two different methods were used to assess nutrient limitation. First, I used nutrient-diffusing flowerpot techniques [54]–[57] from mid-July to mid-August 1998. In these experiments, agar (20 g/l) was poured into 10 cm diameter clay flowerpots with the drain hole plugged by a rubber stopper coded by treatment with a colored thumbtack. The agar either contained no added nutrients (control treatment), 5.0 M NaNO_3_ (N-addition treatment), or 0.5 M NaH_2_PO_4_ (P-addition treatment). After the agar solidified, the pot was sealed at the top using a plastic petri dish attached with silicone cement around the perimeter. Pots were placed in the river with the petri-dish facing down, and were arranged 0.5 m apart in a triangular pattern with the control pot at the upstream apex and the two nutrient addition pots slightly downstream and adjacent to each other, an arrangement which minimizes the chances of nutrient addition treatments affecting other pots. In this experiment, nutrients diffuse through the agar, then through the clay sides of the pot, and become available to algae growing on the pot surface. After 1 month, the pots were removed and algae scraped from the entire pot side with a toothbrush. Algae were stored in 100 ml whirlpack bags with 0.1 ml formalin added as preservative. Upon arrival in the lab, the samples were brought to constant volume, homogenized with a hand blender, and filtered onto CF/C filter paper. Ash free dry mass was measured from the filtered samples as described above for algal biomass on tiles. Nutrient samples taken from covered buckets in the laboratory containing incubating flowerpots confirmed that significant nutrient release continued throughout the duration of the experiment (26.6 mol/d N, 15.2 mol/d P at day 35).

Although nutrient-diffusing flowerpot studies have successfully demonstrated nutrient limitation of periphyton [55]–[58], they may inaccurately portray the extent of nutrient limitation because grazers have access to the pots and may compensate for differences in algal production, and because the clay of the pots may contain residual P. Therefore, in 2001 I also used an alternative technique, measuring short-term algal accumulation on porous glass discs with different nutrient diffusion treatments [58]–[60]. A 35 ml black 35 mm film canister was filled with agar containing either no nutrients (control), 0.5 M NaNO_3_ (N-addition treatment), or 0.05 M NaH_2_PO_4_ (P-addition treatment), and a 2.75 cm diameter porous glass disk, manufactured as a crucible cover (Leco Corporation), was placed on top of the solidified agar. The assembly was held in place with the film canister lid, which had a 2.54 cm hole punched in the center to allow algal colonization and water exchange. In this design, nutrients diffuse through the agar and flow through the porous glass disk, becoming available to algae growing in the interstices of the disk. Canisters were buried in the river bottom with their tops even with the adjacent cobbles, and retrieved after 15–18 days in early August. By permitting algal growth in the interstices of the porous disc and by running the experiment for a short interval, losses to grazing and sloughing are minimized with this design, providing an estimate of algal production under different nutrient conditions. Chlorophyll was extracted from the retrieved discs in 10 ml of 90% ethanol in light-tight film canisters stored in a −20°C freezer for 24 hours, and the resulting extract was analyzed for chlorophyll content using a Turner fluorometer with 430 nm narrow band excitation filter and a 665 nm emission filter. Pure Chl *a* derived from spinach extract (Sigma Chemical) was used as the standard. Because the focus of this study was to determine general trends in nutrient limitation patterns across land use regimes and river downstream location, rather than determining whether any particular river was nutrient limited, a single replicate of each nutrient limitation treatment was placed at each experimental site. If a problem was detected for a particular experiment (e.g., tipped or missing flowerpot, buried or lost film canister), the complete set of treatments was re-deployed again at that site in a subsequent year. Despite these efforts, the +N treatment was lost in both attempts to conduct the porous disc nutrient limitation experiment at the lower South Fork Calawah site, so is missing from the analysis.

### Statistical Analysis

The data were analyzed statistically using a split-plot design ANOVA. In this model, havesting effects were assessed by comparing rivers (random effect) flowing through different land use categories, while downstream location effects (fixed effect) were assessed by comparing sites within rivers. For the analysis, I fit the model,
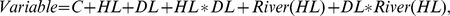
Where *C* is a constant (1 df), *HL* is timber harvest level (2 df), *DL* is downstream location (1 df), HL*DL is their interaction (2 df), *River(HL)* indicates river nested within harvest level (6 df), and DL*River(HL) indicates an interaction of this effect with downstream location (6 df). This model is fully saturated (df = 18). To isolate effects of harvest level, variance due to *River(HL)* was used as the error term and compared to variance due to *HL*. To isolate effects of downstream location and its interaction with harvest level, the DL*River(HL) term was the appropriate error term. All analyses were performed in Systat 12.0.

Sampling sites were treated as the experimental units; therefore when multiple measurements of a variable were taken across years, I analyzed the average value of all measurements from each site. Where necessary to improve the homoscedasticity and normality of residuals, data were log transformed (all biological variables, phosphate, silicate, gradient, and all discharge-related variables). To simultaneously explore the compositional response and overall abundance of aquatic invertebrates, I used a split-plot MANOVA with the densities of Ephemeroptera, Diptera, Plecoptera, and Trichoptera as multiple dependent variables, applying univariate ANOVA for each taxon if overall significant differences were found. I also first applied MANOVA to analyze river width, watershed area and dischrage per watershed area, as discharge per area is derived in part from the others, so would not respond independently. For the analysis of nutrient limitation experiments, nutrient treatment effects and their interactions with land use and downstream location were analyzed as being blocked within downstream location. Given the general interest in factors affecting salmon populations, I also analyzed Pearson correlation coefficients between juvenile salmonids and other variables. Because this was an exploratory analysis involving many pairwise comparisons, correlation coefficients of sufficient size to be considered statistically significant are not considered tests of hypotheses, but indicators of interesting future research directions.

Study sites were chosen based on map information rather than inspection of the river to avoid a biased selection of sites. Inevitably, different rivers have unique geomorphological features that may potentially have strong effects on the response variables measured. Two of the rivers selected in this study had particularly notable features. First, a 20 m high waterfall blocked access of migrating salmonids to the upper Sitkum site. Therefore, fish density response to land use and downstream location was analyzed without this site. Second, during the summer the North Fork Calawah River subsides such that it flows exclusively underground for nearly 13 km, beginning <100 m above the upstream sampling site, despite having reasonably high discharge at the sampling site and no large tributaries in the intervening reach. Therefore, data from these sites were scrutinized as possible outliers and the analyses were redone after omitting the sites if necessary.
